# Critical Analysis of Tools for Measuring Recovery-Oriented Practice in Mental Health Facilities: A Scoping Review

**DOI:** 10.3390/clinpract14060181

**Published:** 2024-10-31

**Authors:** Josè Antonio Garrido-Cervera, María Isabel Ruiz-Granados, Antonio Ignacio Cuesta-Vargas, Antonio José Sánchez-Guarnido

**Affiliations:** 1 Grupo Clinimetría en Fisioterapia (CTS 631), Department of Physiotherapy, Faculty of Health Sciences, University of Málaga, 29071 Málaga, Spain; acuesta@uma.es; 2 Instituto de Investigación Biomédica de Málaga y Plataforma en Nanomedicina (IBIMA Plataforma Bionand) Grupo Clinimetria (F-14), 29590 Málaga, Spain; 3 Department of Physiotherapy, University of Málaga, 29071 Málaga, Spain; 4 Mental Health, Hospital of Antequera, 29200 Málaga, Spain; 5 Department of Psychology, University of Cordoba, 14071 Cordoba, Spain; z22rugrm@uco.es; 6 Mental Health, Hospital Universitario Virgen de las Nieves, 18014 Granada, Spain; antonioj.sanchez.guarnido.sspa@juntadeandalucia.es

**Keywords:** patient-reported experience measures (PREMs), mental health, recovery-oriented services, psychometric properties

## Abstract

Background: To implement recovery-oriented practice, it is important to have instruments capable of evaluating such practice. A number of different questionnaires have been developed in recent years which measure recovery orientation in mental health services. Objective: To identify and analyze patient-reported experience measures (PREMs) reported in the literature that are related to recovery-oriented practice in mental health services. Methodology: This study followed the Joanna Briggs Institute methodology for scoping reviews. Searches were carried out in the Web of Science, CINAHL, Medline (via Pubmed), and SCOPUS databases and in grey literature repositories (Google Scholar, Opengrey, Dart-Europe, Teseo). Papers on recovery services for adults suffering from mental disorders (MDs) were included. Those focusing on addiction and intellectual disability care services were excluded. Results: Sixteen papers met the inclusion criteria. The selected PREMs mainly identified recovery-oriented systems, treatment, community integration and support as the dimensions addressed most frequently in questionnaires. The average number of items included in the questionnaires was found to be 54. With regard to psychometric properties, 62% of the papers evaluated reliability (internal consistency) and 56% provided some kind of evidence of validity. Conclusions: This review aims to give an overview of the existing instruments in the literature and to highlight the characteristics of each one of them. Several different PREMs exist which evaluate recovery-oriented practice. No instrument currently exists which could be described as a benchmark tool, but there are quite a few with good psychometric properties capable of producing data that are useful when evaluating clinical services.

## 1. Introduction

The recovery model has become a guiding concept for mental health service practice [1,2]. However, the implementation of recovery-oriented practice has proved to be a challenge for such services. A range of different organizations are currently working to define and create guiding principles and procedures with which to incorporate this type of approach into daily clinical practice.

Recovery-oriented practice has represented a paradigm change in the therapeutic treatment of people with mental disorders (MDs). Instead of focusing on the illness, it focuses on patients themselves and on their values, the objective being to empower them to lead meaningful, satisfying lives [3,4,5].

Recovery-oriented care is now a consolidated theoretical–philosophical and practical approach for the treatment of people with mental disorders. A proliferation of theoretical models have made it possible to define the main characteristics of recovery, but the practical implementation of such models in mental health services still constitutes a challenge [6].

Two of the difficulties involved are the lack of consensus regarding the actual meaning of recovery-oriented practice and the complexity of transposing theoretical concepts into clinical practice. These challenges have raised a series of questions, such as the following. How can we identify a recovery-oriented mental health service? How can we assess its impact? What benefits does it have for people with mental disorders [7,8,9]?

One example of how the recovery model can be implemented in practice is the CHIME approach, which focuses on five key elements: (C) connectedness, (H) hope, (I) identity, (M) meaning and (E) empowerment. However, this approach has been criticized for being overly optimistic and not taking into account difficulties that may arise during the recovery process. This has led to the proposal of a new approach known as CHIME-D, which includes a sixth dimension: difficulties [10].

Another good basis for incorporating the recovery model into mental health services is the document entitled “Implementing Recovery: A New Framework for Organisational Change” [11], which establishes ten organizational principles for recovery-oriented mental health services: (1) changing the nature of personal interactions; (2) delivering programs led by people with mental illness; (3) developing education centers; (4) creating a “recovery culture”; (5) increasing personal autonomy and decision making; (6) changing approaches to risk assessment and management; (7) redefining user involvement, to increase participation by people with mental illnesses; (8) recovery training for professionals; (9) supporting staff in their recovery journey; and (10) building a life “beyond illness”.

One real, practical example of the implementation of the recovery model has been the creation of Recovery Colleges. Here, an education-based approach is used to support mental health recovery by means of a shared learning, co-production environment. The impact of the Recovery Colleges is twofold; they help people become experts on their own personal care, and at the same time, they help organizations and services focus on the recovery approach. The setting up of recovery-oriented services requires a change in objectives and in the specialist–patient relationship. This type of service focuses not on minimizing symptoms but on rebuilding people’s lives. This implies a conceptual change whereby people with mental disorders come to consider themselves experts in their own personal care while health professionals become their partners in the recovery process [12].

From the examples reported, the recovery model can be seen to have been implemented more extensively in community services, with studies suggesting clear benefits for people with mental disorders [13]. Evidence from hospital environments, however, is more limited [14].

To implement recovery-oriented practices, it is also important to have instruments capable of evaluating such practices. In recent years, a number of different questionnaires have been developed. Several reviews have been published of these questionnaires, aimed at answering three key questions. How is recovery-oriented practice measured in mental health services? What is the current situation with regard to recovery-oriented practice in mental health services? What are the psychometric properties of the different measuring instruments [15]?

Earlier reviews looking at the measurement of recovery-oriented practice have yielded disparate results, with some analyzing only three instruments and others analyzing as many as eighteen. In general, though, they all agree that there is no benchmark measurement and it is therefore difficult to recommend a specific instrument. The most widely used scales are the Recovery Self-Assessment-Revised (RSA-R) and the Recovery Enhancing Environment (REE) instruments [7,16].

Earlier reviews also identified the following limitations in tools for measuring recovery-oriented practice: cultural and geographical adaptation (existing instruments need to be adapted to different cultural and geographical realities), consensus regarding the concept of recovery (many instruments evaluate the same concept from the perspective of very different domains), psychometric properties (many instruments offer insufficient evidence of their psychometric properties), incorporation into clinical practice (questionnaires measuring the extent to which a service is recovery-oriented are not incorporated enough into clinical practice), and adherence to the CHIME conceptual approach (many measurements are not well suited to the CHIME approach) [4,17].

The reviews themselves, however, also have their own limitations. Firstly, they offer no in-depth analysis of the questionnaires reported in the literature. More specifically, they provide no detailed description of their basic characteristics, their psychometric properties, or their quality. Secondly, the literature on recovery model implementation in mental health services is growing rapidly, and the new ways of measuring and assessing that implementation described in the latest papers are not included in those earlier reviews [18].

In view of these limitations, the present study therefore has as its objective to provide a broad overview of the instruments that are available for measuring recovery-oriented practice in mental health services and assessing their quality.

To this end, the decision was made to use the scoping review methodology, which allows wide coverage of the literature and is flexible enough to be able to adjust inclusion and exclusion criteria. This allowed us to identify the highest possible number of questionnaires available in the literature.

## 2. Materials and Methods

A scoping review of studies was carried out following the guidelines set out in the Joanna Briggs Institute (JBI) reviewers’ manual [19], based on the scoping review procedure proposed by Arksey and O’Malley [20] and updated by Levac [21]. It involved five steps: (1) identification of the research question; (2) identification of relevant studies; (3) selection of studies; (4) data extraction; (5) comparing, summarizing and reporting of the results. This review was reported in accordance with the PRISMA (*Preferred Reporting Items for Systematic reviews and Meta-Analyses*) extension for scoping reviews (PRISMA-ScR) [22] and registered in the Open Science Framework https://osf.io/j4vbd/ in January 2023 [23]. This kind of study required no ethical approval.

### 2.1. Identification of the Research Questions and Objective

How is recovery-oriented practice measured in mental health services? What evaluation instruments exist? What are the characteristics and domains of those instruments? What are their psychometric properties?

### 2.2. Identification of Relevant Studies

A search string was created with terms frequently used in the literature pertaining to the recovery model and measuring instruments. The combinations used were as follows: “Recovery orientation and mental health services”; “ Recovery questionnaire and mental health services”; “Recovery scale and mental health services”; “Psychometric properties and mental health recovery”. Two reviewers (JAGC and AJSG) performed the search in four databases from January to July 2023. The information sources used were as follows: CINAhl, SCOPUS, PUBMED and Web of Science. The search was also carried out in the following grey literature repositories: TESEO, Opengrey, Openthesis and Google Scholar. In the grey literature search, the first 200 items relevant to each search term were retrieved. The languages used were English and Spanish. See Scoping Review Search Strategy (Supplementary Annex S1).

The inclusion criteria were defined as follows, using the population, concept and context (PCC) framework:

#### 2.2.1. Participants

Studies focusing on the assessment of recovery orientation in different mental health services were included. Papers studying individual personal recovery processes, health personnel attitudes to the recovery paradigm, and mental health managers’ attitudes were excluded.

#### 2.2.2. Concept

The complexity of the concept of recovery was defined based on the CHIME model and its 5 components: (C) greater social connectedness, (H) encouragement of hope and optimism, (I) identity transformation of someone stigmatized and in the role of passive patient, (M) development of a new meaning in life, often drawing meaning from mental health experiences, and (E) empowerment and responsibility for self-monitoring of mental health [4,24].

We included studies in the validity, reliability, sensitivity and specificity of measuring instruments.

#### 2.2.3. Context

Studies were included regardless of the country, the culture, the type of service and the health care regime (partial or full hospitalization) in which they were carried out.

**Figure 1:**
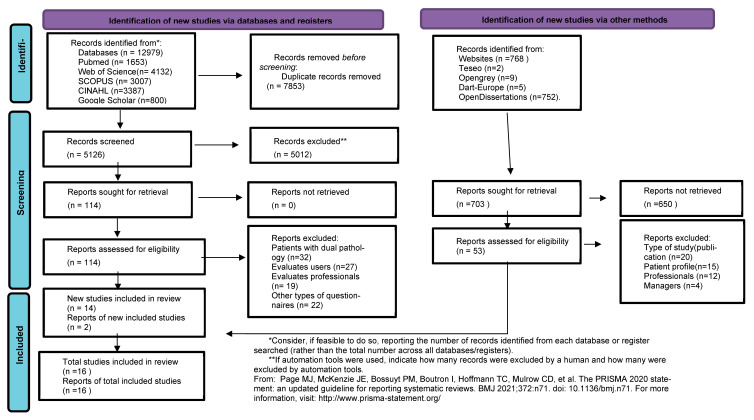
PRISMA 2020 flow diagram for updated systematic reviews which included searches of databases, registers and other sources [25].

For the flow chart diagram, see Figure 1, and for the PRISMA chart, see Supplementary Annex S2.

### 2.3. Selection of Studies

Studies were selected following the PRISMA-ScR flowchart of preferred reporting items for systematic reviews and meta-analysis. The process was conducted by two reviewers (JAGC and AJSG), each of whom selected the relevant abstracts independently. Differences of opinion were resolved through discussion, and when there was no consensus, the decision was referred to a third researcher (AICV).

After excluding those texts which evaluated mental health professionals and mental health service managers, JAG and AJS reviewed the full texts of 16 papers; see Table S1 in the Supplementary Annexes. To assess their methodological quality, all of the studies were critically appraised using the checklist developed by the Joana Briggs Institute (JBI).

### 2.4. Data Extraction

Following Arksey and O’Malley’s stages, the next step was to map the data. In a systematic review, this process is called “data extraction”. The process was carried out by two researchers (JAG and AJS).

### 2.5. Compiling, Summarizing and Reporting of the Data

The data obtained about each scale—including all the most relevant information—were organized into different sections, so that they could then be described and the differences between the different questionnaires analyzed. Verification of the psychometric properties of the questionnaires was based on the COSMIN (Consensus-based Standards for the selection of health Measurement Instruments) tool [26]. This tool uses 10 standards to evaluate the methodological quality of studies that have designed and validated measuring instruments.

## 3. Results

Table 1 and Table 2 detail the main characteristics of the 16 questionnaires selected in this scoping review, including name of instrument, acronym, creators, country of origin, year of publication, type of service, duration and method of administration, domains, number of items, type of measurement, and psychometric values. The instruments’ most noteworthy characteristics are described below.

### 3.1. The Questionnaires’ Countries of Reference

The United States is the country with the biggest scientific output, with 12 questionnaires. Other countries with significant scientific output are the United Kingdom, Australia and Ireland.

### 3.2. Domains of the Personal Recovery Instruments

The most common domains appraised in the service recovery orientation measurement questionnaires were recovery-oriented systems, (eight questionnaires), treatment (six questionnaires), community integration (five questionnaires), support (five questionnaires), services (four questionnaires), participation and meeting basic needs (three questionnaires).

### 3.3. Number of Items in the Questionnaires

The number of items in each questionnaire varied widely, from 166 to just 12. The average number was 52.

### 3.4. Year of Publication

The earliest service recovery orientation measurement questionnaire was published in 2003, and the most recent in 2018. The number of publications was seen to increase over the years, the peak years being 2005, when three questionnaires were published, and 2010, when four questionnaires were published.

### 3.5. Duration and Method of Administration

Most of the service recovery orientation measurement questionnaires use Likert scales. Most of them (14 out of 16) are also self-administered; that is to say, they are filled out by the users themselves. The time taken to complete the questionnaires varies greatly, from 7 to 60 min. The average time required is 20–25 min. However, some authors provide no information about how long their questionnaires take to complete.

### 3.6. Psychometric Properties

The information obtained about the psychometric properties of the service recovery orientation measurement questionnaires is heterogeneous in terms of quality, quantity and type of data.

#### 3.6.1. Reliability

With regard to reliability, most of the questionnaires report good internal consistency based on Cronbach’s alpha analysis. The questionnaires that provide this information are AACP-ROSE, CEO-CRM, EFRS, INSPIRE, REE, RIQ, ROSA, ROSI, RPFS and RSA-R. Only RPFS provides information about test–retest reliability.

#### 3.6.2. Validity

The questionnaires that provide some information about their construct validity (convergent/divergent and discriminant, concurrent or predictive) are AACP-ROSE, CEO-CRM, INSPIRE, MRCRC, REE, ROPI, ROSA, ROSI, RPFS, RSA-R and SRI.

## 4. Discussion

The main objective of this scoping study was to identify, describe and analyze available measuring instruments for assessing the recovery orientation of mental health systems and services.

Recovery-oriented practice requires health systems, services, health professionals, social workers and service users to change the way they look at mental health, and to move from a symptom-based biomedical approach to an approach centered on the individual person and their recovery. In this regard, measuring instruments constitute not only a key tool for establishing and defining the results obtained but also a useful guide when attempting to improve care [27].

Broadly speaking, there exists a wide range of measuring instruments that can be used to evaluate recovery orientation in mental health services. This is positive, because it reflects a growing interest in the recovery paradigm [15,16,28].

With regard to the questions posed in this study, most of the questionnaires analyzed can be described as quantitative, Likert-type instruments that are self-administered by the patients themselves. However, some characteristics are more heterogeneous. The number of items, for example, ranges from 14 to 166, depending on the purpose for which the questionnaire was created.

The most commonly evaluated domains in the recovery orientation measurement instruments analyzed were recovery-oriented systems, treatment, community integration and support. These domains are essential to the recovery paradigm since they reflect its most fundamental objectives. Recovery focuses on the ability of people with mental disorders to achieve their goals and live full, meaningful lives despite their limitations. It is evaluated by means of items that measure things like self-determination, self-care, emotional well-being and social participation. Treatment focuses on the availability of effective, accessible treatment services for people with mental disorders, and is evaluated using items that measure things like service quality, service accessibility, and users’ satisfaction with the services they receive. Community integration focuses on the ability of people with mental disorders to participate in their community and cultivate meaningful relationships. This is evaluated using items that measure things like participation in community activities, social relationships and participation in the employment market. Support focuses on the availability of social and emotional support for people with mental disorders, and is evaluated through items that measure things like family support, friends’ support, and support received from health care professionals. Evaluation of these domains can help mental health services improve their recovery orientation [28,29]. Moreover, this is important when discussing the support domains and the role of services in preventing relapse and rehospitalization [30].

The psychometric properties of the recovery orientation measurement instruments analyzed were heterogeneous. Some instruments had been exhaustively validated, while others had undergone no psychometric assessment whatsoever. Reliability was in general acceptable. In other words, the scores obtained by these instruments were relatively consistent, although further research is required to assess their validity [31].

Improvements to the reliability and validity of these recovery orientation measurement instruments can potentially have a positive impact on the quality of mental health services. The instruments can be used to appraise the progress of people with mental disorders, compare different mental health services, and identify the areas in which such services can be improved.

In short, interest in the evaluation of recovery orientation in mental health services can be said to have grown over the last few years. The different PREMs that are available with which to assess recovery orientation vary with regard to the aspects they evaluate, the type of questions they use and their target audiences. Each one has its own pros and cons, and it is important to take these factors into account when choosing which recovery orientation evaluation tool to use. Some instruments may need to be adapted, or to have additional studies carried out before they can be used. It may also be necessary to develop new instruments with which to evaluate recovery orientation in specific contexts [15,23,28,32].

This review makes a useful contribution in the field of recovery orientation evaluation thanks to three main strengths: its use of the scoping review methodology, the high number of questionnaires analyzed, and the amount of information provided. By adhering to a scoping review framework, the study was able to explore newly emerging evidence related to this topic, including grey literature. This is a strength insofar as grey literature tends to be an important source of information about emerging or under-researched subjects. Also, the analysis of 16 questionnaires enabled the authors to offer a broad overview of available recovery orientation evaluation tools, allowing readers to compare and contrast different instruments and choose the one that best meets their needs. Finally, detailed information is provided about all of the questionnaires analyzed, including their objectives, the domains they evaluate and their psychometric properties. This is a strength because it gives readers in-depth knowledge of each instrument.

Like all scoping reviews, this study is not without its limitations. Firstly, it would have been useful to create and register a protocol beforehand, to be able to pre-plan the paper and identify possible variations between the protocol and the final review. Secondly, the lack of a universally accepted definition of recovery and its associated reliability criteria meant that the way the term was used was variable. Nevertheless, we attempted to broadly include all questionnaires encompassing dimensions of recovery as their point of focus. Third, the search was restricted exclusively to instruments in two languages—English and Spanish. This could limit the applicability of findings from studies in other languages. Fourth, it is impossible to discriminate whether the instruments are more suitable for use in a hospital or community setting. Finally, we consider it necessary to develop and generate new measuring instruments, adapted to the different cultures and values of each country.

Future research might focus on adapting and validating questionnaires for other countries, to allow them to be used in different cultural and linguistic contexts. It would also be important to carry out more research into instrument validity, in order to improve our understanding of the psychometric properties of different tools. It would be interesting to evaluate which tools are more suitable for which environments (hospital, community). Another aspect to consider would be the comparability between different assessment tools and their sensitivity to change to detect changes in the recovery process between different groups of patients. Another question to investigate could be how treatment quality and accessibility affect patient satisfaction and long-term outcomes in recovery-oriented models [33].

In summary, the evaluation of recovery-oriented practice still lacks a “Gold Standard” reference measure. However, instruments such as INSPIRE and RSA stand out due to their comprehensiveness, strong psychometric properties (reliability and validity), brief administration time (4 and 10 min, respectively), and ease of understanding. Additionally, the categories of both instruments align with the domains of the CHIME recovery model. Finally, it is important to highlight that the RSA is the most widely used and validated instrument across diverse populations and countries.

## 5. Conclusions

In recent years, several instruments have been developed to evaluate recovery-oriented clinical practice in mental health services. These PREMs can be useful in encouraging, monitoring and improving the quality of such services. Their use, however, is still not widespread in clinical practice.

The authors agree with other researchers that greater consensus is needed regarding what the concept of recovery actually means. This would make it possible to determine the most suitable instruments for evaluating recovery orientation in mental health services [16,28,29,30].

To conclude, the authors consider that the use of such instruments for evaluating recovery-oriented practice needs to become more widespread in both research and clinical environments.

## Figures and Tables

**Table 1:** Instruments we have identified, in alphabetical order, are as follows.

Instrument	Acronyms
American Association of Community Psychiatrists Recovery Oriented Services Evaluation	(AACP-ROSE)
Consumer Evaluation of the Collaborative Recovery Model	(CEO-CRM)
Elements of a Recovery Facilitating System	(EFRS)
INSPIRE	INSPIRE
Magellan Recovery Culture Report Card	(MRCRC)
Pillars of recovery service audit Tool	(PORSAT)
Recovery Based Program Inventory	(RBPI)
Recovery Enhancing Environment Measure	(REE)
Recovery Interventions Questionnaire	(RIQ)
Recovery Oriented Practices Index	(ROPI)
Recovery Oriented Services Assessment	(ROSA)
Recovery Oriented Systems Indicators Measure	(ROSI)
Recovery Promotion Fidelity Scale	(RPFS)
Recovery Self-Assement	(RSA-R)
Implementing Recovery Oriented Services	(SAPT)
Scottish Recovery Indicator	(SRI)

**Table 2:** Characteristics of the evaluation instrument devices.

Instrument	Acronym	Authors	Country of Origin	Year	Type of Devices	Time and Method of Administration	Domains	No. of Items	Type of Measurement	Reliability	Validity	Sensitivity and Specificity
American Association of Community Psychiatrists Recovery Oriented Services Evaluation	AACP-ROSE	American Association of Community Psychiatrists	United States	2010	Organization promoting guidance	8–10 minCan be completed by people in recovery (people receiving services), managers, family members and clinicians	Administration Treatment Supports Organizational Culture	46	5-point Likert scale	Internal consistency (Cronbach’s alpha = 0.98)	Not specified	Not specified
Consumer Evaluation of the Collaborative Recovery Model	CEO-CRM	Marshall et al.	United States	2009	Mental health services	Not specifiedSelf-Administered (Service User or Case Manager)	recovery-oriented systems	14	7-point Likert scale	Internal consistency (Cronbach’s alpha = 0.78–0.80)	Apparent validityContained validity	Not specified
Elements of a Recovery Facilitating System	EFRS	Ridgway	United States	2007	Recovery-oriented mental health services	Not specifiedUser and/or user’s family	person-centered consumer-driven community-focused accessible/integrated	20	5-point Likert scale	Internal consistency (Cronbach’s alpha = 0.96)	Not specified	Not specified
INSPIRE	INSPIRE	Mike Slade	United Kingdom	2010	Personal recovery support services	7 minUser	connectedness Hope Identity Meaning and purpose Empowerment	21	5-point Likert scale	Internal consistency (Cronbach’s alpha =0.82–0.85)	Content validityConstruct validity	Specified
Magellan Recovery Culture Report Card	MRCRC	Burgess et al.	United States	2010	Recovery-based culture	Not specified User and/or Family member and/or Supervisor	welcoming and accessibility growth orientation consumer inclusion emotionally healing environments and relationships quality of life focus community integration staff morale and recovery	102	Questions	Notspecified	Construct validityDiscriminant validity	Not specified
Pillars of recovery service audit Tool	PORSAT	Agnes Higgins	Ireland	2008	Determine whether services are in line with scale domains	Not specified User and/or family member and/or Provider	Leadership Person-centered and empowering care Hope-inspiring relationships Access and inclusion Education Research/Audit	60	4-point Likert scale	Not specified	Not specified	Not specified
Recovery Based Program Inventory	RBPI	Ragins, M.	United States	2004	Recovery orientation	Not specified Mental health professionals	Recovery Beliefs and Implementation Recovery Relationship and Leadership recovery culture recovery treatment	148	Qualitative	Not specified	Not specified	Not specified
Recovery Enhancing Environment Measure	REE	Ridgway y Press	United States	2004	Evaluating the recovery model	30–45 minAdult user	Demographics data Stage of Recovery importance Ratings Elements of Recovery services; Special Needs of the individual Organizational Climate Recovery Markers Consumer Feedback	166	5-point Likert scale	Internal consistency (Cronbach’s alpha =0.94–0.97)	Apparent validityContained validity Concurrent validity	Not specified
Recovery Interventions Questionnaire	RIQ	Ellis and King	Australia	2003	Recovery orientation	Not specified Service users and case managers.	Aspects of support treatment which facilitates recovery	50	5-point Likert scale	Internal consistency (Cronbach’s alpha =0.64	Not specified	Not specified
Recovery Oriented practices Index	ROPI	Mancini AD and Finnerty MT	United States	2005	Measure recovery-oriented practices at the organizational level.	Not specifiedUser	meeting basic needs comprehensive services customization and choice consumer involvement/participation network supports/community integration strengths-based approach client source of control/self-determination recovery focus	20	5-point Likert scale	Notspecified	Content validityConstruct validity	Not specified
Recovery-Oriented Services Assessment	ROSA	Amy C. Lodge, Wendy Kuhn, Juli Earley, and Stacey Stevens Manser	United States	2018	Recovery-oriented service evaluation	Not specified Service users	recovery-oriented systems	15	5-point Likert scale	Internal consistency (Cronbach’s alpha = 0.92–0.95)	Not specified	Not specified
Recovery Oriented Systems Indicators Measure	ROSI	Dumont, J.M., Ridgway, P., Onken, S.J., Dornan, D.H., and Ralph, R.O	United States	2005	Evaluate the recovery orientation of community mental health systems for adults with severe and prolonged psychiatric disorders.	30 min User and Professionals	Adult Consumer Self-Report Survey Person-centered decisionmaking and choice Invalidated personhood Self-care and wellness Basic life resources Meaningful activities androles Peer advocacy Staff treatment knowledge Access Administrative Data Profile Peer support Choice Staffing ratios System culture and orientation Consumer inclusion in governance Coercion	42 usersAdministration 23	Short, 5-point Likert-type questions	Internal consistency (Cronbach’s alpha = 0.95)	Apparent validity Contained validity	Not specified
Recovery Promotion Fidelity Scale	RPFS	Armstrong NP and Steffen JJ.	United States	2009	Assessing the fidelity of services to recovery-oriented practices	Not specifiedUser	collaboration participation and acceptance self-determination and peer support quality improvement development	12	5-point Likert scale	Internal consistency (Cronbach’s alpha) = 0.98/0.97/0.95Test–Retest: ICC = 0.72/0.72/0.75	apparent Validity Validity content	Not specified
Recovery Self- Assessment	RSA-R	O’Connell et al.	United States	2005	Evaluate the recovery-oriented practices of mental health services	10 minPerson in recovery; family member; mental health professional	life goals involvement diversity of treatment options choice individually tailored services	36	5-point Likert scale	Internal consistency (Cronbach’s alpha = 0.76–0.90)	Apparent validity Contained validityValidity Construct validity	Not specified
Implementing Recovery-Oriented Services	SAPT	James Winarski, M.S.W. Michael Dow, Ph.D. Patrick Hendry Patricia Robinson, Ph.D.	United States	2018	Assess the degree of implementation of recovery-oriented services	Not specifiedService users	Administration Treatment Community Integration	50	4-point Likert scale	Not specified	Not specified	Not specified
Scottish Recovery Indicator	SRI	Anthony Mancini	Scotland	2010	Recovery orientation	20 minService users	Meeting basic needs Personalization and choice Strengths-based approach Comprehensive service Service-user involvement/participation Involving support networks and promoting social inclusion and community integration Service user in control and active participant even when subject to compulsion Recovery focus	20	Qualitative (Questions)	Not specified	Construct validity	Not specified

## Data Availability

The data presented in this study are available on request from the corresponding author. The data are not publicly available because they are part of an ongoing project.

## Supplementary Materials

The following supporting information can be downloaded at: https://www.mdpi.com/article/10.3390/clinpract14060181/s1, Supplementary Annex S1. Scoping Review Search Strategy. Supplementary Annex S2. Preferred Reporting Items for Systematic reviews and Meta-Analyses extension for Scoping Reviews (PRISMA-ScR) Checklist.
