# Supplementary material for: Critical Analysis of Tools for Measuring Recovery-Oriented Practice in Mental Health Facilities: A Scoping Review
Source: Clin Pract. 2024 Oct 31;14(6):2313–28. doi: 10.3390/clinpract14060181 (PMC11586944; doi:10.3390/clinpract14060181)
Supplement: Supplementary file 1 [file clinpract-14-00181-s001.zip › Annex S1. .pdf]

Scoping Reviews

Search Strategy Template

Database name (platform name) – date searched
PubMed (U.S. National Library of Medicine, National Institutes of Health) – July 1, 2023

Search	Query	Items Found
1	(("recoveries"[All Fields] OR "recovery"[All Fields]) AND ("orient"[All Fields] OR "orientability"[All Fields] OR "orientable"[All Fields] OR "orientate"[All Fields] OR "orientated"[All Fields] OR "orientates"[All Fields] OR "orientating"[All Fields] OR "orientation"[MeSH Terms] OR "orientation"[All Fields] OR "orientations"[All Fields] OR "orientation s"[All Fields] OR "orientation, spatial"[MeSH Terms] OR ("orientation"[All Fields] AND "spatial"[All Fields]) OR "spatial orientation"[All Fields] OR "oriented"[All Fields] OR "orientational"[All Fields] OR "orienting"[All Fields] OR "orients"[All Fields]) AND ("mental health services"[MeSH Terms] OR ("mental"[All Fields] AND "health"[All Fields] AND "services"[All Fields]) OR "mental health services"[All Fields])) AND (alladult[Filter])	377
2	(("recoveries"[All Fields] OR "recovery"[All Fields]) AND ("questionnair"[All Fields] OR "questionnaire s"[All Fields] OR "surveys and questionnaires"[MeSH Terms] OR ("surveys"[All Fields] AND "questionnaires"[All Fields]) OR "surveys and questionnaires"[All Fields] OR "questionnaire"[All Fields] OR "questionnaires"[All Fields]) AND ("mental health services"[MeSH Terms] OR ("mental"[All Fields] AND "health"[All Fields] AND "services"[All Fields]) OR "mental health services"[All Fields])) AND (alladult[Filter])	725
3	(("recoveries"[All Fields] OR "recovery"[All Fields]) AND ("scale s"[All Fields] OR "scaled"[All Fields] OR "scaling"[All Fields] OR "scalings"[All Fields] OR "weights and measures"[MeSH Terms] OR ("weights"[All Fields] AND "measures"[All Fields]) OR "weights and measures"[All Fields] OR "scale"[All Fields] OR "scales"[All Fields]) AND ("mental health services"[MeSH Terms] OR ("mental"[All Fields] AND "health"[All Fields] AND "services"[All Fields]) OR "mental health services"[All Fields])) AND ((1980/1/1:2022/7/1[pdat]) AND (alladult[Filter]))	448
4	("psychometrical"[All Fields] OR "psychometrically"[All Fields] OR "psychometrics"[MeSH Terms] OR "psychometrics"[All Fields] OR "psychometric"[All Fields]) AND ("properties"[All Fields] OR "property"[All Fields]) AND ("mental health recovery"[MeSH Terms] OR ("mental"[All Fields] AND "health"[All Fields] AND "recovery"[All Fields]) OR "mental health recovery"[All Fields])) AND ((1980/1/1:2022/7/1[pdat]) AND (alladult[Filter])	103
5	AND ("1980"[Date - Publication]: "2022"[Date - Publication]) AND English[Language] AND SPANISH [Language]	1653

2

Database name (platform name) – date searched
Web Of Sciencie- July, 1, 2023

Search	Query	Items Found
1	"Recovery orientation and mental health service"	350
2	"Recovery questionnaire and mental health services"	1232
3	"Recovery scale and mental health services"	1434
4	"Psychometric properties and mental health recovery"	516
5	AND ("1980"[Date - Publication]: "2023"[Date - Publication]) AND English[Language] AND SPANISH [Language]	4132

Database name (platform name) – date searched
SCOPUS July, 1, 2023

Search	Query	Items Found
1	(recovery AND orientation) AND TITLE-ABS-KEY (mental AND health AND services)) AND (LIMIT-TO (LANGUAGE , "English") OR LIMIT-TO (LANGUAGE , "Spanish")	292

Scoping Reviews

2	(recovery AND questionnaire) AND TITLE-ABS-KEY (mental AND health AND services)) AND (LIMIT-TO (LANGUAGE , "English") OR LIMIT-TO (LANGUAGE , "Spanish"))	973
3	(recovery AND scale) AND TITLE-ABS-KEY (mental AND health AND services)) AND (LIMIT-TO (LANGUAGE , "English") OR LIMIT-TO (LANGUAGE , "Spanish"))	945
4	(psychometric AND properties) AND TITLE-ABS-KEY (mental AND health AND services)) AND (LIMIT-TO (LANGUAGE , "English") OR LIMIT-TO (LANGUAGE , "Spanish"))	797
5	AND ("1980"[Date - Publication]: "2023"[Date - Publication]) AND English[Language] AND SPANISH [Language]	3007

Database name (platform name) – date searched v
CINAHL-EBSCOhost- July, 1, 2022
3

Search	Query	Items Found
1	recovery orientation AND mental health services	651
2	recovery questionnaire AND mental health services	224
3	recovery scale AND mental health services	385
4	Psychometric properties AND mental health services	2154
5	AND ("1980"[Date - Publication]: "2023"[Date - Publication]) AND English[Language] AND SPANISH [Language]	3387

Other Searching Methods

Searching Completed (Yes/No)	Searching Method	Date Searched	Items Found
TESEO, OPRENGREY, Dart-Europe, OpenDissertations	Backward Citation Searching	1970-2023	703
